# Cardiovascular disease and asymptomatic childhood cancer survivors: Current clinical practice

**DOI:** 10.1002/cam4.3190

**Published:** 2020-06-18

**Authors:** Wendy J. Bottinor, Debra L. Friedman, Thomas D. Ryan, Li Wang, Chang Yu, Scott C. Borinstein, Justin Godown

**Affiliations:** ^1^ Division of Cardiovascular Medicine Department of Medicine Vanderbilt University School of Medicine Nashville TN USA; ^2^ Department of Pediatrics Division of Hematology‐Oncology Vanderbilt University Medical Center Nashville TN USA; ^3^ Department of Pediatrics University of Cincinnati College of Medicine; Heart Institute Cincinnati Children's Hospital Medical Center Cincinnati OH USA; ^4^ Department of Biostatistics Vanderbilt University Medical Center Nashville TN USA; ^5^ Department of Pediatrics Division of Pediatric Cardiology Vanderbilt University Medical Center Nashville TN USA

**Keywords:** cardiovascular diseases, heart failure, referral and consultation, surveys and questionnaires, survivors, survivorship

## Abstract

**Background:**

It is poorly understood how cardiovascular screening in asymptomatic childhood cancer survivors (CCS) is applied to and impacts clinical care.

**Objectives:**

To describe the current role of cardiovascular screening in the clinical care of asymptomatic CCS.

**Methods:**

At 50 pediatric academic medical centers, a childhood cancer survivorship clinic director, pediatric cardiologist, and adult cardiologist with a focus on CCS were identified and invited to participate in a survey. Surveys were managed electronically. Categorical data were analyzed using nonparametric methods.

**Results:**

Of the 95 (63%) respondents, 39% were survivorship practitioners, and 61% were cardiologists. Eighty‐eight percent of survivorship practitioners reported that greater than half of CCS received cardiovascular screening. CCS followed by adult cardiology were more likely to be seen by a cardio‐oncologist. Those followed by pediatric cardiology were more likely to be seen by a heart failure/transplant specialist. Common reasons for referral to cardiology were abnormal cardiovascular imaging or concerns a CCS was at high risk for cardiovascular disease. Ninety‐two percent of cardiologists initiated angiotensin converting enzyme inhibitor or angiotensin receptor blocker therapy for mild systolic dysfunction. Adult cardiologists initiated beta‐blocker therapy for less severe systolic dysfunction compared to pediatric cardiologists (*P* < .001). Pediatric cardiologists initiated mineralocorticoid therapy for less severe systolic dysfunction compared to adult cardiologists (*P* = .025). Practitioners (93%) support a multi‐institutional collaboration to standardize cardiovascular care for CCS.

**Conclusions:**

While there is much common ground in the clinical approach to CCS, heterogeneity is evident. This highlights the need for cohesive, multi‐institutional, standardized approaches to cardiovascular management in CCS.

## Graphical abstract

The implementation and impact of cardiovascular screening recommendations in asymptomatic childhood cancer survivors is characterized based on survey responses from 50 academic medical centers. Heterogeneity in current clinical practice highlights the need for multidisciplinary alliances, standardization of care across institutions, and collaborative research programs to improve evidence‐based care for this population.

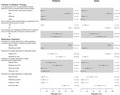

## 1 INTRODUCTION

Cardiovascular disease, a recognized complication of specific cancer therapies, such as anthracyclines, platinum agents, and radiation therapy, affects a significant portion of childhood cancer survivors (CCS).^1^, ^2^, ^3^ Due to the magnitude of the problem, it is recommended that asymptomatic CCS identified as high risk undergo serial cardiovascular screening.^4^, ^5^, ^6^, ^7^, ^8^ Within this population, however, the optimal screening frequency and modality have not been clearly defined.^9^, ^10^, ^11^ In addition, the efficacy of medical therapy in asymptomatic CCS is not well established and is an active area of investigation.^9^, ^12^, ^13^, ^14^, ^15^

Given the limited data regarding the optimal approach to cardiovascular screening and treatment in asymptomatic CCS, we hypothesized that clinical management in this population is likely heterogenous. Our goal, therefore, was to describe current practice patterns and approaches to the management of asymptomatic CCS, using a sample of academic medical centers with established pediatric oncology programs. We describe current patterns among these institutions with respect to cardiovascular screening, cardiology referral patterns, practice setting, thresholds for initiating medical treatment, and classes of medications used in the treatment of asymptomatic CCS.

## 2 METHODS

This study is a cross‐sectional survey of practice patterns for cardiovascular screening and management among providers who care for CCS. This study was reviewed and approved by the Institutional Review Board (IRB). Based on IRB review, a separate ethics committee approval was not recommended. An electronic survey was developed in collaboration with experts from the Survey Research Shared Resource Center at our institution. The survey was reviewed by practitioners with expertise in pediatric oncology and cancer survivorship, pediatric cardio‐oncology, pediatric advanced heart failure/cardiac transplant, and adult cardio‐oncology to ensure questions were clear and appropriate for each of the specialties surveyed. The survey was designed differently for oncology practitioners and cardiologists.

A priori, the top 50 academic medical centers, with nationally recognized pediatric hematology/oncology programs, were identified by using US News and World Report rankings.^16^ At each institution, three practitioners, a survivorship clinic director, pediatric cardio‐oncologist, and an adult cardio‐oncologist, were identified using the respective health system's professional website. If a cardio‐oncologist could not be identified, an advanced heart failure/transplant practitioner or the division chair was invited to complete the survey. Invitees were encouraged to forward the survey to a colleague if they believed a more appropriate respondent was available at their institution. Research Electronic Data Capture (REDCap) was used for survey distribution and data collection. Survey responses were collected anonymously and respondents were assured that no institutional‐level data would be disclosed.

### 2.1 Statistical analysis

Responses from practitioners who reported caring for both pediatric and adult CCS were included in both pediatric and adult analyses. Three respondents checked both yes and no for one question each. These three ambiguous responses were excluded from analysis.

Survey responses are presented as frequency (percentage). Between‐group comparisons for categorical data were made using Fisher's exact test. Statistical analysis was performed using R Statistical Software (R Foundation, Vienna, Austria).

## 3 RESULTS

A total of 50 pediatric hematology/oncology programs within academic medical centers were identified. Initial invitations were sent to 152 practitioners. Among survivorship clinic directors, 53 initial invitations were sent because two programs had clinical co‐directors and one program had separate clinics for pediatric and adult survivors of childhood onset malignancy. Of note, for one institution, a survivorship clinic director could not be identified; therefore, a survey invitation was sent to the pediatric hematology/oncology division chief. A total of 50 initial invitations were sent to pediatric cardiologists. Initial invitations were sent to 49 adult cardiologists because a corresponding adult‐trained cardiologist could not be identified for one pediatric hematology/oncology program. The initial invitees forwarded the survey to eight additional practitioners.

Ninety‐five practitioners (63%) responded to the survey. Of these respondents, 37 (39%) were survivorship practitioners and 58 (61%) were cardiologists. Among survivorship practitioners, 19 (51%) primarily cared for pediatric age survivors, 17 (46%) cared for both pediatric and adult age survivors, and 1 (3%) cared for primarily adult age childhood cancer survivors. Among cardiologists, 27 (47%) cared for pediatric age survivors, 7 (12%) managed both pediatric and adult age survivors, and 24 (41%) focused their practice on adult age childhood cancer survivors (Table 1).

**Table 1:** Characteristics of survey respondents: specialty and primary patient population

	Percent (N)
Cardiology	58
Pediatric Cardiology	47% (27)
Adult Cardiology	41% (24)
Pediatric and Adult Cardiology	12% (7)
Survivorship	37
Pediatric Survivorship	51% (19)
Adult Survivorship	3% (1)
Pediatric and Adult Survivorship	46% (17)

### 3.1 Survivorship

Eighty‐eight percent of survivorship practitioners estimated that > 50% of the CCS in their practice undergo screening at the intervals recommended by Children's Oncology Group or other guidelines. The most common reason for referring a CCS to a cardiologist was abnormal cardiac imaging (91%). At total of 15% of respondents also reported that increased risk for cardiovascular disease was a common reason for referral. Most (71%) survivorship practitioners reported a specific cardiologist or group of cardiologists who focus on CCS were available at their institution. Almost all (96%) of respondents stated they would be interested in developing a multi‐institutional consortium to standardize referrals and care for CCS.

### 3.2 Cardiology

#### 3.2.1 Cardiovascular screening and referral patterns

**Figure 1:**
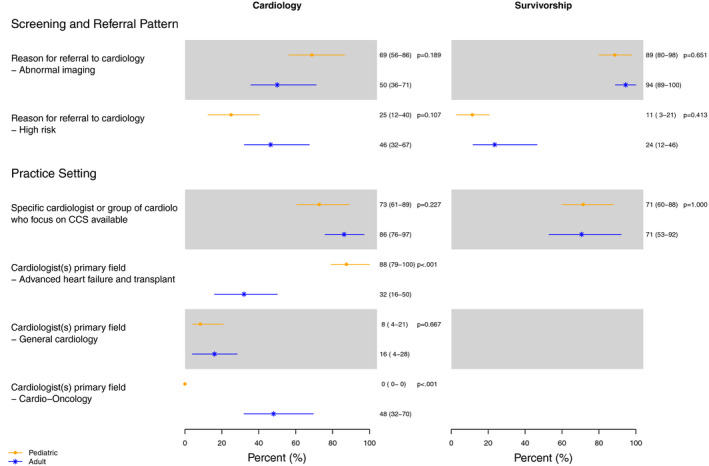
Screening and Referral Patterns: A comparison of screening and referral patterns among cardiologists and survivorship practitioners. The most common reasons for referral were abnormal cardiac imaging or perception of a survivor as high risk for cardiovascular disease. The primary field of training varied among adult and pediatric cardiologists. P values represent comparisons between pediatric and adult providers. Values are expressed as percentage (95% CI)

The most common reasons for referral were abnormal imaging and identification of a survivor as high risk for cardiovascular disease. Specifically, 50% [95% CI: 36, 71%] of adult cardiologists and 69% [95% CI: 56, 86%] of pediatric cardiologists identified abnormal imaging as the most common reason for referral (*P* = .189). Forty six percent of adult cardiologists [95% CI: 32, 67%] and 25% [95% CI: 12, 40%] of pediatric cardiologists reported identification of a survivor as high risk for cardiovascular disease as the most common reason for referral (*P* = .107) (Figure 1).

#### 3.2.2 Practice setting

Eighty‐six percent [95% CI: 76, 97%] of adult cardiologists and 73% [95% CI: 61, 89%] of pediatric cardiologists reported a specific cardiologist or group of cardiologists who focus on CCS were available at their institution (Figure 1). In most adult focused practices, the primary field of practitioners caring for CCS was cardio‐oncology (48% [95% CI: 32, 70%]). In most pediatric practices, the primary field of practitioners caring for CCS was advanced heart failure/transplant (88% [95% CI: 79, 100%]) (Figure 1).

#### 3.2.3 Cardiovascular imaging

Seventy‐nine percent of cardiologists, when faced with any abnormal cardiac screening study, reported they would first repeat cardiac imaging. The most common modalities used for repeat imaging were echocardiography and cardiac magnetic resonance imaging.

#### 3.2.4 Initiation of medical therapy

Most cardiologists (86% [95% CI: 79, 100%] of adult providers and 85% [95% CI: 76, 96%] of pediatric providers) identified mild systolic dysfunction as their threshold for initiating medical therapy. A total of 10% of respondents also reported initiating medical therapy in CCS with normal function but low LV mass.

**Figure 2:**
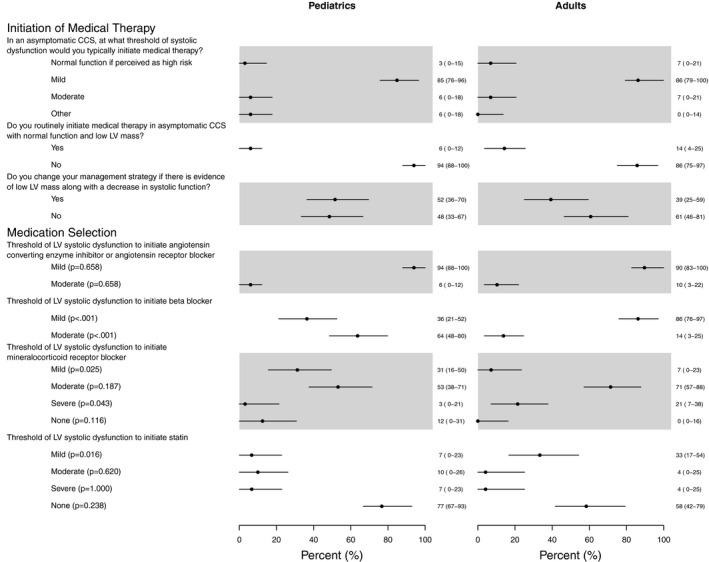
Threshold for Initiation of Medical Therapy: A comparison of adult of pediatric cardiology thresholds for medication initiation. Almost all providers identified mild left ventricular systolic dysfunction as the threshold for initiating medical therapy. Thresholds for specific classes of medications varied depending on whether the provider was an adult of pediatric cardiologist. *P* values represent comparisons between pediatric and adult providers. Values are expressed as percentage (95% CI). CCS = childhood cancer survivor, LV = Left ventricular

Structural changes also influenced management. Fifty‐two percent [95% CI: 36, 70%] of pediatric cardiologists reported that low left ventricular mass in addition to systolic dysfunction would change their management strategy while 61% [95% CI: 46, 81%] of adult cardiologists reported that the low left ventricular mass in the setting of systolic dysfunction would not change their management strategy (Figure 2).

#### 3.2.5 Medication selection

Pharmacotherapeutic management practices demonstrated variability based on the cardiologist's primary population (adult vs pediatrics). Most (92%) initiate angiotensin‐converting enzyme inhibitors (ACEi)/angiotensin receptor blockers (ARB) for mild systolic dysfunction. More adult cardiologists initiate beta‐blockers for mild systolic dysfunction compared to pediatric cardiologists, 86% [95% CI: 76, 97%] vs 36% [95% CI: 21, 52%], *P* < .001. While most cardiologists initiate mineralocorticoid therapy for moderate dysfunction, 71% [95% CI: 57, 88%] among adults and 53% [95% CI: 38, 71%] among pediatric cardiologists, *P* = .187, more pediatric cardiologists initiate mineralocorticoid therapy for mild dysfunction compared to adults cardiologists, 31% [95% CI: 16, 50%] vs 7% [95% CI: 0, 23%], *P* = .025. Statin therapy for secondary prevention was not commonly used among either group of cardiologists; however, 42% of adult cardiologists would recommend statin therapy for some CCS while 77% of pediatric cardiologists do not recommend statin therapy (Figure 2).

#### 3.2.6 Additional testing

**Figure 3:**
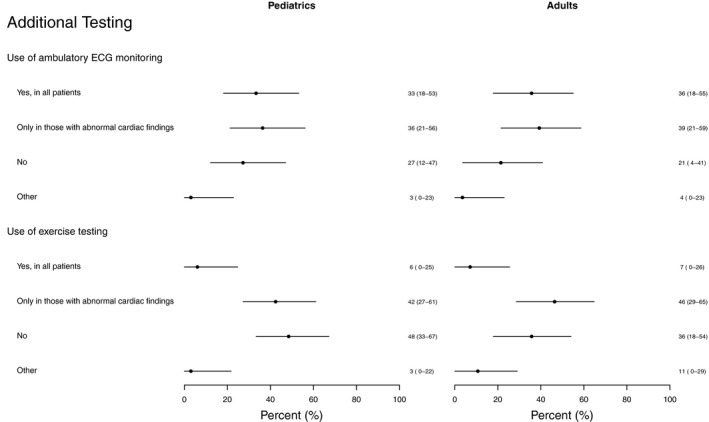
Use of Additional Testing: A comparison of adult of pediatric cardiology thresholds for additional testing. Responses regarding the use of additional testing were particularly heterogeneous among cardiologists. Values are expressed as percentage (95% CI). ECG = electrocardiogram, percentage (95% CI)

Cardiologists were also asked about their practice patterns regarding ambulatory electrocardiogram (ECG) monitoring and exercise testing. Management in these areas was particularly heterogeneous with an almost equal number of respondents using ambulatory ECG monitoring in all patients (34%), no patients (25%), and only selected patients with abnormal cardiac findings (38%) (Figure 3). Responses were also heterogeneous regarding the use of routine exercise testing in selected patients with abnormal cardiac findings versus no routine use of exercise testing (44% and 43%, respectively) (Figure 3).

#### 3.2.7 Future directions

Among cardiologists, 90% stated they would be interested in developing a multi‐institutional consortium to standardize referrals and care for CCS.

## 4 DISCUSSION

Our results describe current cardiac screening and management practices in asymptomatic CCS cared for at nationally recognized medical centers. These findings suggest cardiovascular screening in asymptomatic CCS is routinely incorporated into clinical practice in centers with established survivorship programs. The most common reasons for cardiology referral and thresholds for initiating medical therapy are similar across institutions, although the proportion of referrals for each of these reasons, practitioner focus (cardio‐oncology vs advanced heart failure/transplant), and threshold for initiating specific classes of medications is more variable. Interest was high among respondents to standardize care for CCS.

Current clinical practice patterns in asymptomatic survivors and the influence of cardiac screening results on clinical management is an understudied area. While a recent study performed in a large academic center found high adherence to screening recommendations, other studies have demonstrated lower rates.^17^, ^18^, ^19^ Marr et al found that less than 10% of CCS followed for a median of 8 years were up‐to‐date with screening recommendations. A dedicated survivorship clinic was noted to increase screening adherence.^19^ This finding may help to explain the much higher estimates of compliance reported by our survey respondents, as we specifically surveyed practitioners working in institutions with established survivorship clinics.

The most common reasons for cardiology referral were abnormal cardiac imaging and increased risk for cardiovascular disease. This finding is supported by recent work from the American College of Cardiology (ACC) Pediatric Cardio‐Oncology Work Group. This group also demonstrated that patients were more likely to be evaluated by a cardiologist after completion of cancer therapy than prior to or during therapy.^20^ It is unclear why adult cardiologists reported more referrals for CCS at increased risk for cardiovascular disease compared to survivorship practitioners or pediatric cardiologists. One potential explanation may be the higher incidence of cardiovascular comorbidities in CCS as they age and the known association between these risk factors and adverse cardiovascular events in this population.^21^

Most pediatric cardiologists reported advanced heart failure/transplant as their primary field in contrast to adult cardiologists for whom almost half identified cardio‐oncology. This difference likely reflects disparities in educational opportunities as there is an absence of dedicated pediatric cardio‐oncology training programs in the United States.^20^, ^22^ It is likely that this is an area of potential need.

Our study also reports current practice patterns regarding medical therapy in asymptomatic CCS in a sample of US institutions with pediatric oncology and survivorship programs. We found heterogeneity in clinical management among these institutions, a finding that has also been observed in international health systems. A study within the Dutch health system found wide variations in the medications and dosing schedules used in asymptomatic CCS with evidence of cardiotoxicity.^23^ The lack of population specific studies showing efficacy of traditional stage B heart failure therapy in this population may explain this heterogeneity and therefore reemphasizes the recognized and ongoing need for further investigation into therapies targeted to CCS.^3^

Practitioners surveyed in our study reported a lower threshold for initiating ACEi/ARB therapy compared to beta‐blocker or mineralocorticoid therapy. This has also been demonstrated by the ACC Pediatric Cardio‐Oncology Work Group.^20^ While expert consensus supports the use of medical therapy in all asymptomatic patients with cardiomyopathy, efficacy data specific to CCS is lacking and in general, management strategies are primarily extrapolated from other populations.^3^, ^24^, ^25^, ^26^, ^27^, ^28^ Given this lack of CCS‐specific efficacy data, practitioners may rely on specialty specific (adult cardiology focused vs pediatric cardiology focused) literature to direct management, and this may account for some of the variability in our findings.

In patients with adult onset malignancy, the use of ACEi/ARB and beta‐blockers appears to be effective in treating anthracycline‐mediated cardiomyopathy.^29^, ^30^, ^31^ However, early detection and therapy appear crucial for the efficacy of these medications. These data from the adult population may explain the lower threshold for initiating beta‐blocker therapy we observed among adult cardiologists.

A beneficial role for ACEi/ARB and beta‐blocker therapy in pediatric populations has been more difficult to demonstrate. In CCS with abnormal left ventricular function, a transient improvement in cardiac function has been demonstrated with ACEi therapy; however, this benefit is lost after approximately 6‐10 years due to progressive left ventricular wall thinning.^12^, ^13^ The role of beta‐blockers for the treatment of anthracycline‐mediated cardiomyopathy in pediatric populations is not well established. Although some evidence supports a prophylactic role, the largest randomized trial of beta‐blocker therapy in pediatric patients with heart failure did not demonstrate a benefit.^32^, ^33^ The use of beta‐blockers in this population is an area of active investigation; however, the current lack of data to support beta‐blocker use in pediatric populations may help to explain the higher threshold for initiating beta‐blocker therapy we observed among pediatric cardiologists.^15^

Data to support the use of statins in cancer survivors are limited; however, retrospective studies in survivors of adult onset malignancies support a beneficial role.^34^, ^35^ A recent prospective trial in CCS did not support the use of statins; however, this study may have been underpowered.^36^ Although not a statistically significant difference, higher statin use was reported among adult cardiologists. This practice pattern may reflect the supportive data from adult populations.

Cardiac growth and left ventricular mass can be compromised by exposure to anthracyclines. Anthracycline exposure may directly result in lower cardiac mass through inhibition of topoisomerase 2β, potentially leading to endothelial dysfunction and the loss of cardiac progenitor cells.^37^ Indirectly, anthracycline exposure may also lead to cardiotoxic effects through reduction of cardioprotective proteins.^38^ This reduction in left ventricular mass was first described in patients with pediatric onset malignancy, and may help to explain our observation (although not statistically significant) that pediatric cardiologists were more likely to modify their management strategy based on the presence of low left ventricular mass.^39^ Age specific factors, such as the increased hemodynamic demands that occur during adolescence, also likely contribute to the pediatric cardiologist's approach to low LV mass.

Although differences in clinical management were identified, our results do help to identify common practice patterns. These common practices include referral of CCS who are thought to be high risk for the development of cardiovascular disease or are found to have abnormal cardiac imaging. Additionally, the threshold of mild left ventricular systolic dysfunction for the initiation of medical therapy is an area of common clinical management strategy. Moving forward, these areas of common ground can be used to establish a multi‐institutional, standardized referral and clinical management approach for CCS, an endeavor supported by almost all survey respondents and by experts in this field.^20^, ^40^

Limitations of our study include the potential for introducing selection bias by surveying only academic medical centers with highly ranked pediatric oncology programs. All programs surveyed have a dedicated survivorship clinic, which has been shown to improve adherence to long‐term surveillance, and therefore responses may not reflect all health systems. In addition, selecting only programs which meet US News and World Report criteria for high ranking may also create selection bias. Our reliance on self‐reported data may introduce recall or observational biases. It is also possible that practitioners who participated may be different than those who did not participate in the survey. Our study was conducted approximately 1 year after the Children's Oncology Group version 5 guidelines were released. This relatively recent change in guidelines may also have influenced responses.^4^ Lastly, while we can identify common practice patterns, these data do not identify best practices and it is possible that less common practices in fact optimize patient outcomes.

## 5 CONCLUSIONS

We describe current clinical practice patterns for the cardiovascular care of CCS and the influence of cardiac screening results on clinical management. Our results identify an interest among practitioners in standardizing care for CCS and current areas of commonality related to referral patterns and treatment approach. While we did find some heterogeneity in terms of clinical approach, much of this heterogeneity appears to be centered around the practitioner's primary specialty (adult vs pediatric cardiology). These findings emphasize the need for multi‐institutional collaboration and further investigative research into the optimal management of cardiovascular health in CCS.

## CONFLICT OF INTEREST

The authors declare no conflicts of interest relevant to this research.

## AUTHORS’ CONTRIBUTIONS

Wendy J Bottinor: conceptualization, data curation, formal analysis, funding acquisition, methodology, writing—original draft, and writing—review and editing. Debra L Friedman: conceptualization, investigation, methodology, supervision, visualization, writing—review and editing. Thomas D Ryan: investigation, validation, visualization, writing—review and editing. Li Wang: formal analysis, methodology, validation, and writing—review and editing. Chang Yu: formal analysis, methodology, validation, and writing—review and editing. Scott C Borinstein: conceptualization, investigation, methodology, resources, supervision, visualization, and writing—review and editing. Justin Godown: conceptualization, data curation, formal analysis, investigation, methodology, resources, supervision, validation, visualization, writing—original draft, and writing—review and editing.
